# Synthesis of Holmium-Oxide Nanoparticles for Near-Infrared Imaging and Dye-Photodegradation

**DOI:** 10.3390/molecules27113522

**Published:** 2022-05-30

**Authors:** Jia Zhu, Xue-Jun Shao, Zongan Li, Chia-Hui Lin, Cheng-Wan-Qian Wang, Keran Jiao, Jian Xu, Hong-Xia Pan, Ye Wu

**Affiliations:** 1 Department of Mechatronic Engineering, Suzhou City University, Suzhou 215000, China; jia.zhu@szcu.edu.cn; 2 Department of Clinical Laboratory Medicine, Children’s Hospital of Soochow University, Suzhou 215025, China; xjshao@suda.edu.cn; 3 Jiangsu Key Laboratory of 3D Printing Equipment and Manufacturing, School of Electrical and Automation Engineering, Nanjing Normal University, Nanjing 210046, China; zongan_li@njnu.edu.cn; 4 Suzhou Gallant Biotech Biotechnology Co., Ltd., Suzhou 215000, China; calacolin@gpmbio.cn; 5 Department of Clinical Laboratory Medicine, Suzhou BenQ Medical Center, Affiliated BenQ Hospital of Nanjing Medical University, Suzhou 215000, China; wanqian_1019@163.com; 6 Department of Chemistry, Xi’an Jiaotong-Liverpool University, Suzhou 215000, China; keran.jiao16@student.xjtlu.edu.cn; 7 Division of Electrical and Computer Engineering, Louisiana State University, Baton Rouge, LA 70803, USA

**Keywords:** holmium oxides, near-infrared imaging, fluorescence imaging, dye-photodegradation, nanoparticles

## Abstract

The development of multifunctional nanomaterials has received growing research interest, thanks to its ability to combine multiple properties for severing highly demanding purposes. In this work, holmium oxide nanoparticles are synthesized and characterized by various tools including XRD, XPS, and TEM. These nanoparticles are found to emit near-infrared fluorescence (800–1100 nm) under a 785 nm excitation source. Imaging of the animal tissues was demonstrated, and the maximum imaging depth was found to be 2.2 cm. The synthesized nanoparticles also show the capability of facilitating dye (fluorescein sodium salt and rhodamine 6G) degradation under white light irradiation. The synthesized holmium oxide nanoparticles are envisioned to be useful for near-infrared tissue imaging and dye-degradation.

## 1. Introduction

Fluorescence microscopy has been widely used in daily clinic operations for organ tissue inspection. One of the major technical challenges in microscopy-based fluorescence deep-tissue imaging is the serious light scattering, that results in vague images. Research efforts from different aspects have been devoted to overcoming this technical hurdle [1,2,3,4,5]. Among these methods, a promising solution is to use new fluorescent agents that can minimize the light scattering, such as metal sulfide compounds [6], carbon-based compounds [7], polymers [8], proteins [9], Au-Cu-Se heterodimer compounds [10], metal nanoparticles [11], Zn-Ga-Sn compounds phosphors [12], CdSe-ZnS-polymers compounds [13], InGaP-ZnS-chitosan compounds nanoparticles [14], CdTe-CdSe-ZnSe core-shell-shell nanostructures [15], β-NaGdF_4_-based upconversion phosphors [16], hybrid quantum dot-fatty ester stealth nanoparticles [17], LiGa_5_O_8_-based nanoparticles [18], novel metal nanoclusters [19], NaYF_4_-based upconversion phosphors [20], CdSeS-ZnS-organic com-pounds quantum dots [21], metal-organic compounds [22], small-molecule [23], and NaYbF_4_-based Core/Shell nanoparticles [24]. It should be noted that using polymeric or organic material systems to synthesize fluorescent agents can bring additional drawbacks. Some of these fluorescent agents have been previously reported to show very broad emission spectra that make them hard distinguish from the background fluorescence, as there are nonlinear interactions between the light wave and the materials [24]. Additionally, they are usually limited by photostability and blinking emission effect [24].

Metal-based compounds are an alternative. In these materials, the ligands around the metal ions will absorb the light energy, which is then transferred to the metal ions. The emission is thus initiated from the metal ions. As various metal ions can produce different fluorescence bands, the targeted spectra can be precisely engineered. The synthesis of the metal-based compounds is very flexible given that there are many choices for the metal ions. The making of near-infrared (NIR) imaging contrasts is important. NIR optical wavelengths range (880–2200 nm) can lead to better tissue imaging quality, compared to the visible wavelengths, thanks to reduced optical scattering and low optical absorption. Furthermore, imaging depth in centimeters can be acquired that is otherwise difficult to be achieved within the ultraviolet-visible region [5,12]. The optical emission of metal-based compounds can be particularly adjusted via NIR emissive metal ions including Pr^3+^, Gd^3+^, Dy^3+^, Er^3+^, Nd^3+^, Y^3+^, La^3+^, and Yb^3+^ [25,26,27].

Among all suitable metal-based compounds for being as NIR imaging contrasts, holmium-oxide nanoparticles have recently emerged as one of the promising candidates, thanks to their facile preparation method, excellent tunability for wavelength-calibration calibration tools, and good potential for acting as the pyrolysis catalysts [28,29,30]. Moreover, holmium-oxide nanoparticles also possess good photocatalytic properties for degrading the common water pollutants [31,32], which makes holmium-oxide nanoparticles also a promising candidate for reducing the current severe water pollution problems. Though the dual-functionalities of holmium-oxide nanoparticles have attracted significant research interest and some research advances have been made in terms of synthesis method and application usage, using holmium-oxide nanoparticles for degradation of fluorescein sodium salt and rhodamine 6G under white light irradiation as well as NIR imaging of animal tissue, to the best of our knowledge, has not yet been reported.

Herein, we report the synthesis of holmium-oxide nanoparticles and demonstrate their dual-functionalities in terms of NIR tissue imaging and photocatalyst. The synthesis method is facile and requires less than 5 h, and no sophisticated equipment and labware are needed. They were characterized by transmission electron microscope (TEM), X-ray photoelectron spectroscopy (XPS), and X-ray powder diffraction (XRD) technology. NIR fluorescence in the range of 800–1100 nm was observed. Their application in NIR deep tissue was tested by using pig bacon meat tissues. The imaging depth of 2.2 cm beneath the tissue surface was achieved. In addition, holmium-oxide nanoparticles were found to be useful for photodegradation of rhodamine 6G and fluorescein sodium salt under white light irradiation, which is rarely reported in the current literature. We envision that the simple chemistry, facile synthesis, and dual-functional performance make the holmium oxide compound nanoparticles attractive for bio-imaging and organic-pollutants processing.

## 2. Materials and Methods

### 2.1. Materials

Fumaric acid, 2-methylimidazole, dimethylsulfoxide, l-cysteine, sulfuric acid, holmium oxide, fluorescence sodium salt, and rhodamine 6G were all purchased from Alfa Aesar (Haverhill, MA, USA).

### 2.2. Preparation of Ho-Oxide Compound Nanoparticles

In the first step, we prepared three kinds of organic solutions: Solution 1, Solution 2, and Solution 3. Solution 1 was made by dissolving 2-methylimidazole (2 g), terephthalic acid (5 g), fumaric acid (5 g), d (+)-glucose (5 g), oxalic acid dehydrate (5 g), dimethyl sulfoxide (50 mL) in deionized water (200 mL). Solution 2 was prepared by mixing di-methylformamide (300 mL), oleic acid (40 mL), triethylamine (40 mL), diethylene glycol (40 mL), dimethyl sulfoxide (20 mL) and methacrylic anhydride (10 mL). Solution 3 was prepared by dissolving l-cysteine (2 g) in deionized water (50 mL). Then Solution 1, Solution 2, and Solution 3 were mixed, the light-yellow precipitate appeared accordingly. The mixture solution was stirred for 3–4 h to obtain a clear solution (Solution 4). For the synthesis of the Ho-oxide compound, a solution containing Ho ions was first prepared. Holmium (III) oxide (5 g) and sulfuric acid (20 mL) were mixed and stirred, then deionized water (50 mL) was added drop by drop. The acquired pink solution was heated to a temperature of 100 ℃ to obtain a clear Solution 5. Solution 5 was mixed with Solution 4, then this mixture was stirred for 2 h to obtain Solution 6 containing the Ho-oxide compound. Solution 6 was firstly centrifuged with a speed of 6000 RPM for 0.5 h, then 10,000 RPM for 0.5 h, followed by 14,000 RPM for 0.5 h to obtain the final Ho-oxide compound nanoparticles.

### 2.3. Characterization

The XPS data were collected via a K-Alpha XPS instrument (Thermo Scientific, Waltham, MA, USA). Powder XRD characterization data was collected via a PANalytical Empyrean XRD instrument. TEM images were taken by using a JEOL 1400 TEM (120 kV) (Tokyo, Japan). The UV-Vis-NIR absorption was collected through a SPARK spectrometer (Tecan, Grodig, Austria).

### 2.4. NIR Fluorescence Measurement and NIR Tissue Imaging Setup

**Figure 1:**
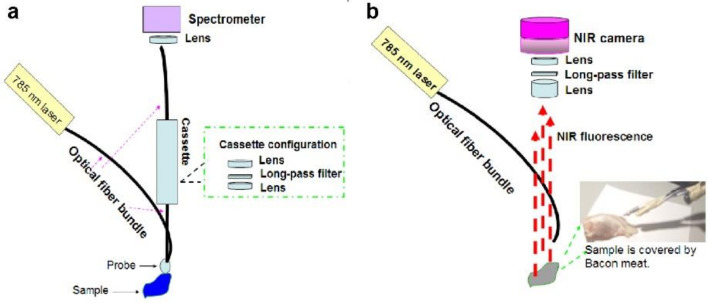
(**a**) Schematic for measurement of NIR fluorescence. (**b**) Schematic for NIR tissue imaging.

Two experimental facilities were used for the measurement of fluorescence. The first facility was a commercial SPARK fluorescence spectra meter, which was used to measure the fluorescence with respect to the excitation wavelength of 280 nm, 380 nm, 480 nm, 580 nm, and 680 nm. The second one is a self-built fluorescence setup (Figure 1a), which provides the excitation wavelength of 785 nm and the NIR fluorescence collection from 800 nm to 1100 nm. As shown in Figure 1a, a laser diode (Thorlab Inc., Newton, NJ, USA) with a center wavelength of 785 nm was used as the excitation source. Two optical fiber bundles with a diameter of 3 mm were used. One was used to deliver the excitation light, and another one was used for the collection of the fluorescence. They were combined in a single probe, which was moved around the surface of the sample to collect the fluorescence. Given that the excitation light can be also collected by the optical fiber bundle, we used an NIR long-pass filter (Thorlab Inc.) to eliminate the excitation light. The filter was set up between the lens and seated inside a cassette. The collected fluorescence was measured by a spectrometer (Ocean Optics, Dunedin, FL, USA).

Another facility was built to investigate the potential application of these compounds in tissue imaging (Figure 1b). A 785 nm laser is used as an excitation light source. An optical fiber bundle is applied to deliver the optical power. When the sample surface is shined by the laser, NIR fluorescence can emit from the sample surface. The image of the sample can be captured by a NIR camera seated above the sample. A centrifuge tube filled with Ho-oxide compound nanoparticles in dimethylformamide (2 mL) solution was used as the imaging object. The centrifuge tube was first put in the position around the NIR camera to take a control image. Then, the centrifuge tube was covered with layers of bacon meat. The NIR camera was applied again to check the image of the centrifuge tube and evaluate imaging depth. Although the bacon meat can block some of the NIR fluorescence emitting from Ho-oxide compound nanoparticles, some of the strong NIR fluorescence can still penetrate through bacon meat and reach the NIR camera. If more and more layers of bacon meat were used, the image of the centrifuge tube captured in the NIR camera began to blur and fade. Here commercial pig bacon meat was used as sample tissues to carry out an experiment since pig bacon meat is similar to human body tissue.

## 3. Results and Discussion

### 3.1. TEM, XRD and XPS Characterization

For characterization of the holmium oxide (Ho) nanoparticles, transmission electron microscopy (TEM), X-ray powder diffraction (XRD), and X-ray photoelectron spectroscopy (XPS) were used. TEM was used to investigate the size of the synthesized nanoparticles and the typical images are shown in Figure 1a. The average diameter of the Ho-oxide compound is found to be 25 nm based on 10 TEM images.

**Figure 2:**
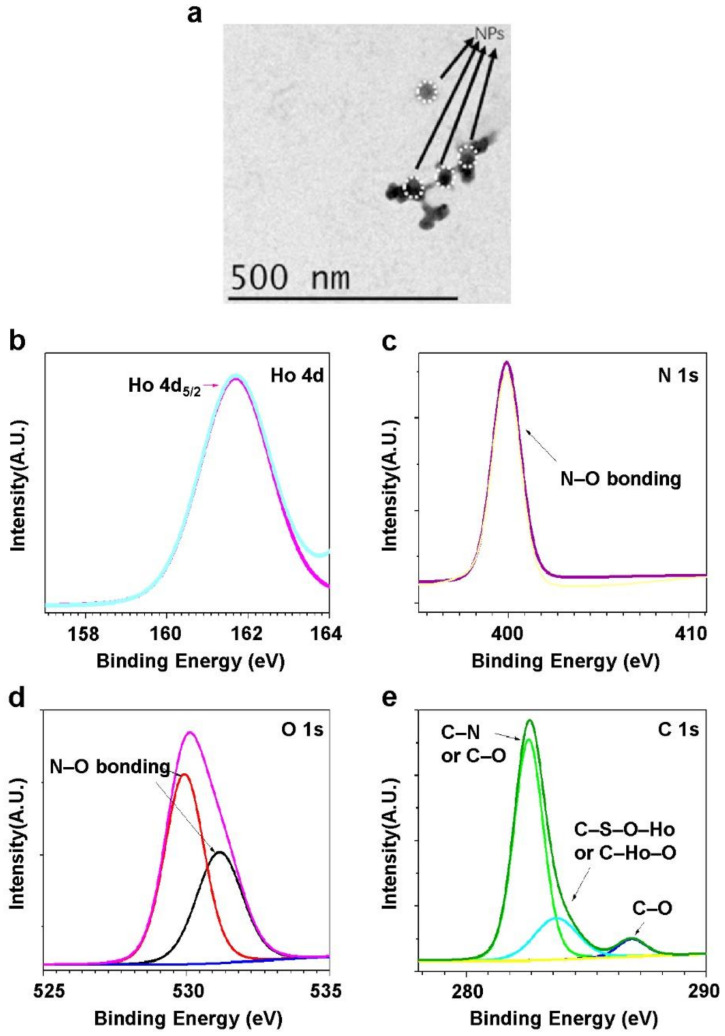
(**a**) Typical TEM image of the Ho-oxide nanoparticles. High resolution of XPS spectra for different elements: (**b**) Ho 4d; (**c**) N 1s; (**d**) O 1s; (**e**) C 1s.

High-resolution XPS scanning spectroscopy was carried out for studying the bonding information of the synthesized nanoparticles. Ho 4d, C 1s, N 1s, and O 1s spectra of Ho-oxide compound are clearly seen in Figure 2b–e. The XPS spectra of Ho 4d (Figure 2b) show a peak at 161.62 eV, which is corresponding to Ho 4d 5/2 core level in the Ho-based compound [33,34]. The binding energy of pure Ho 4d is 161.8 eV. The chemical bonding of Ho–O or Ho–O–C–N makes it shift to 161.62 eV [35,36]. The C 1s spectra present three peaks at 282.59 eV, 283.79 eV, and 286.88 eV. The peak at 282.59 eV is assigned to C–N or C–O bonding. The peak at 283.79 eV is considered C–N–O–Ho or C–Ho–O bonding. The peak at 286.88 eV is attributed to C–O bonding [37]. N 1s spectra reveal a peak at 399.89 eV, which is attributed to N–O bonding [38]. O 1s spectra show peaks at 529.92 eV and 531.13 eV, which are considered as N–O bonding [38]. XRD was also applied to study the crystal structure of the materials (see Figure S1 of Supplementary Materials for phase indexing and lattice parameters). Other material characterization results such as energy dispersive X-ray and ultraviolet-visible spectroscopy can be found in other articles [29,39].

### 3.2. Optical Physics

**Figure 3:**
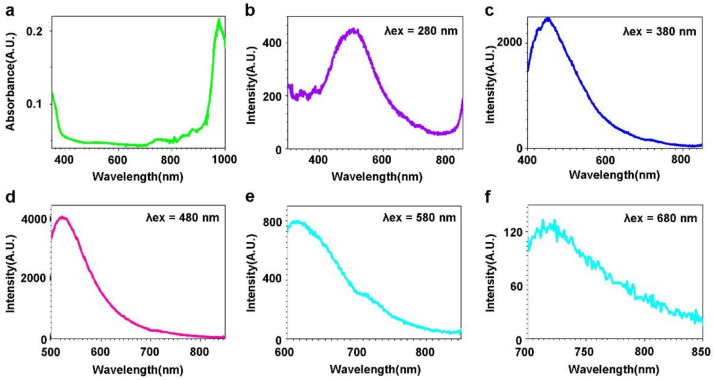
Optical property of Ho-oxide compound. (**a**) Optical absorption is plotted. Fluorescence spectra with different excitation are investigated: (**b**) 280 nm; (**c**) 380 nm; (**d**) 480 nm; (**e**) 580 nm; (**f**) 680 nm.

Excitation sources with different excitation wavelengths of 280 nm, 380 nm, 480 nm, 580 nm, 680 nm, and 785 nm were used to study the optical property of the Ho-oxide compound. The optical absorption spectrum (Figure 3a) of the Ho-oxide compound presents peaks at 739.67 nm, 876.91 nm, and 976.91 nm, and a shoulder at 350–387 nm. The 739.67 nm peak is considered due to the transition from the ^5^I_8_ level to the ^5^I_4_ level. The 876.91 nm peak is corresponding to the transition from the ^5^I_8_ level to the ^5^I_5_ level. The 976.91 nm peak is related to the transition from the ^5^I_8_ level to the ^5^I_6_ level [40,41,42,43].

The fluorescence spectra with excitation light of 280 nm, 380 nm, 480 nm, 580 nm, and 680 nm are shown in Figure 3b–f. Figure 2b presents the fluorescence spectra with the excitation wavelength of 280 nm showing one peak at 507.85 nm. With the excitation wavelength of 380 nm (Figure 3c), the peak of fluorescence spectra of the Ho-oxide compounds is around 450.98 nm. When the excitation wavelength is 480 nm, green light emission is observed with a peak of 520.19 nm (Figure 3d). The 520.19 nm peak corresponds to the transition from the ^5^S_2_ level to the ^5^I_8_ level [41].

**Figure 4:**
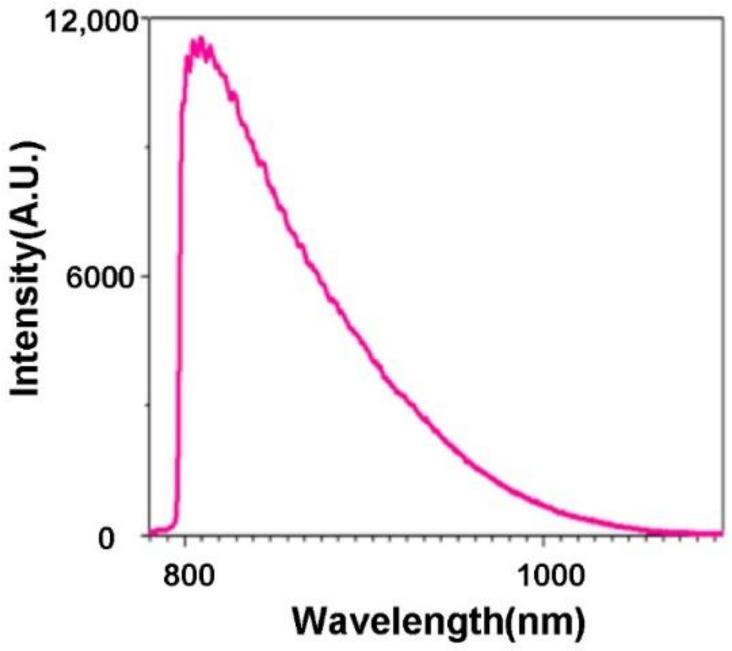
NIR fluorescence of Ho-oxide compound (excitation wavelength = 785 nm).

When an excitation wavelength of 580 nm is applied, red emission is observed, as shown in Figure 3e. The fluorescence spectra of Ho-oxide compound depict a peak at 613.11 nm, which is regarded as the transition from the ^5^F_5_ level to the ^5^I_8_ level [41]. When red light (680 nm) is used as an excitation source, NIR emissions from 700 nm to 850 nm can be observed (Figure 3f). A peak can be found at 722.07 nm, which is related to the transition from the ^5^S_2_ level to the ^5^I_8_ level [41]. As shown in Figure 4, NIR fluorescence from 798 nm to 1100 nm for the compound can be observed. A peak is presented at 809.34 nm.

### 3.3. NIR Tissue Imaging

**Figure 5:**
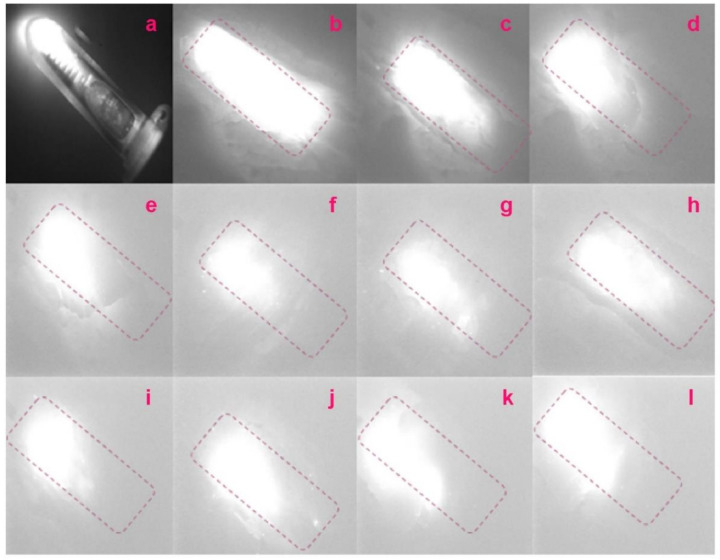
(**a**) Control image is a 2 mL centrifuge tube filled with Ho-oxide compound solution. The image of the tube under several layers of bacon meat: (**b**) one layer; (**c**) two layers; (**d**) three layers; (**e**) four layers; (**f**) five layers; (**g**) six layers; (**h**) seven layers; (**i**) eight layers; (**j**) nine layers; (**k**) ten layers; (**l**) eleven layers. Here, the dotted line purple box is presented to show the profile and edge of the tube. Even covered by 11 layers of the bacon meat whose total thickness is 2.2 cm, we can still see the shape and edge of the tube.

**Figure 6:**
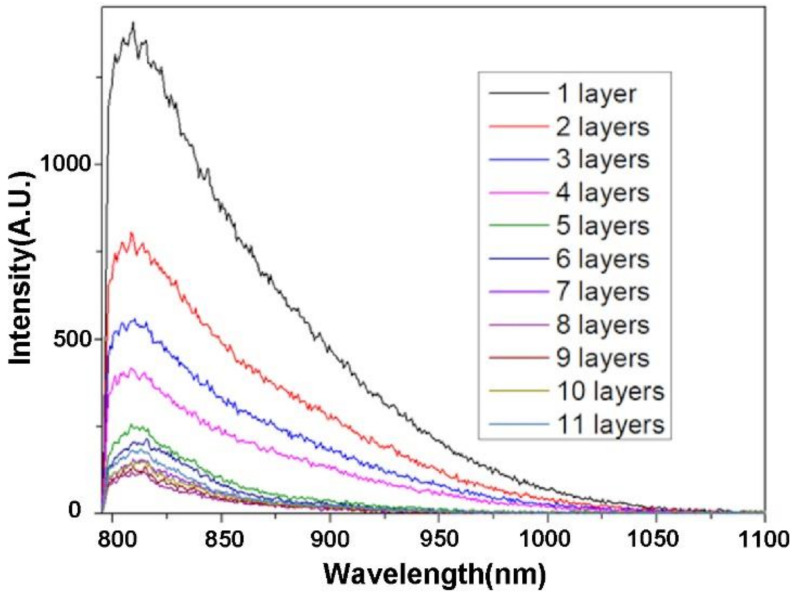
The intensity of the NIR fluorescence of the sample detected was suppressed after covered by various layers of bacon meat.

Figure 5a–i show the tissue imaging results of Ho-oxide compound nanoparticles. Until covering the centrifuge tube with eleven layers of bacon meat, the NIR image of the tube has been suppressed. Considering the thickness of one layer of bacon meat is 2 mm, the depth of eleven layers of bacon meat is 2.2 cm. This is the maximum imaging depth we can achieve for the Ho-oxide compound. Figure 6 indicates that the NIR fluorescence intensity of the experimental tube was suppressed after covering with several layers of bacon meat. When we carried out the tissue imaging experiment, the position of the camera and excitation light was found to be critical. To obtain the correct position, in the beginning, we had to use a white light source (such as a flashlight, etc.) to align the whole optical path. This can save time for optical alignment.

Traditionally, optical coherence tomography (OCT) is ubiquitously used in tissue imaging. However, its imaging depth can only be limited to 1–2 mm below the tissue surface. If 8 the depth is large, the proportion of light that can overcome scattering is too small to be detected. It should be mentioned that the NIR fluorescence from our synthesized nanoparticles is very strong: with that tissue depth of 1–2.2 cm, we can see the object of the centrifuge tube that is filled with those nanoparticles under the tissue. Therefore, the potential application of these nanoparticles in NIR tissue imaging is promising. They can be used as the active image contract agents in biomedical imaging systems. We can expect their combination with certain imaging systems such as endoscopes or optical fiber bundles to be used for dental and medical procedures. They enable the remote and distributed measurement of the morphology of the tissue under the skin.

### 3.4. Effect of Photodegradation

**Figure 7:**
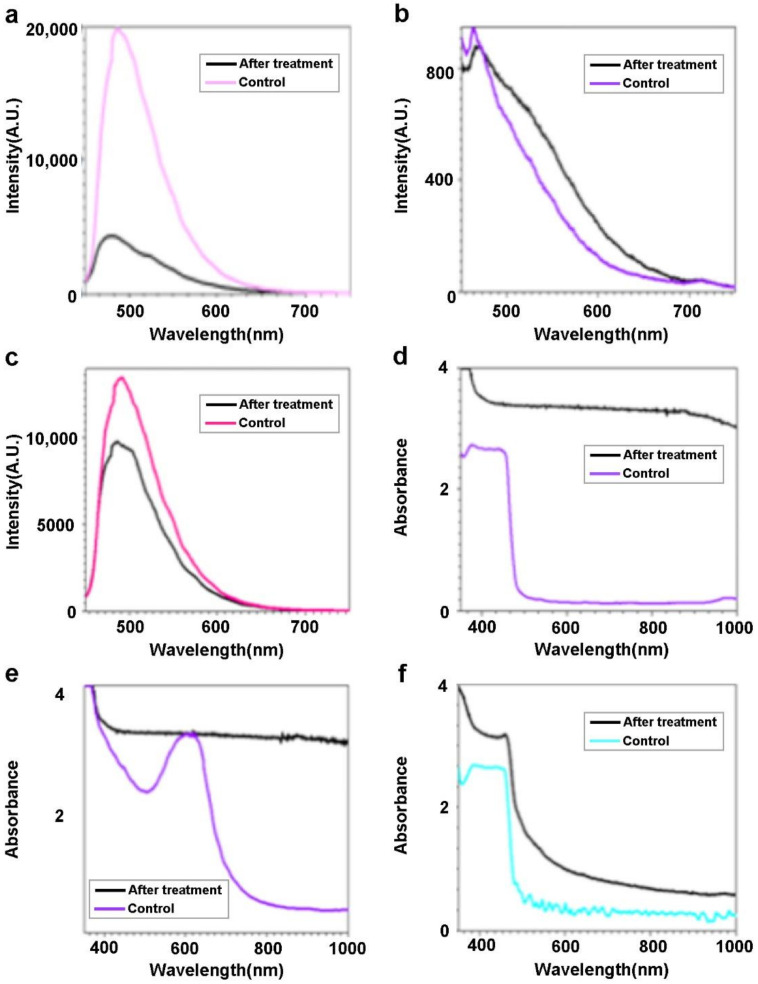
(**a**) Fluorescence of FSS after degradation treatment under white light irradiation. (**b**) Fluorescence of R6G after degradation treatment under white light irradiation. (**c**) Fluorescence of Dye 1 after degradation treatment under white light irradiation. (**d**) Absorption of FSS after degradation treatment under white light irradiation. (**e**) Absorption of R6G after degradation treatment under white light irradiation. (**f**) Absorption of Dye 1 after degradation treatment under white light irradiation.

Photodegradation of dyes has attracted considerable attention from researchers [44,45,46]. We chose two kinds of dyes: fluorescein sodium salt and rhodamine 6G as the dye examples for the photodegradation experiment, as both dyes are commonly used in this kind of experiment, and also, represent the common organic pollution [47,48,49]. Detailed information about dye molecular structure, toxicity, and molecular mass can be found in Table S1. Optical spectra were used to analyze the impact of dye-photodegradation. As seen in Figure 7a, there is a peak at 486.62 nm upon 440 nm light excitation before photodegradation treatment. After photodegradation treatment, the peak is shifted to be at 478.03 nm and its intensity is reduced dramatically. Figure 7b indicates the fluorescence change of R6G due to the impact of dye-photodegradation treatment. Before treatment, the fluorescence shows a peak at 463.54 nm upon 440 nm light excitation. After treatment, it shows a peak at 470.56 nm, whose intensity is 5% less than that of the 463.54 nm peak. Figure 7c presents the change of fluorescence for Dye 1 (mixture of fluorescein sodium salt (FSS) and R6G) after photodegradation treatment. The fluorescence shows a peak at 490.94 nm before treatment. After treatment, the peak is shifted to be at 485.55 nm and its intensity is 27.4% less than that of the 490.94 nm peak.

Optical absorption spectroscopy is applied to examine the impact of photodegradation (Figure 7d–f). Before photodegradation treatment, the absorption spectrum of FSS shows an absorption band around 378 nm–457 nm (Figure 7d). After treatment, this absorption band disappears. An absorption band around 523 nm–669 nm is shown in the absorption of R6G before photodegradation treatment (see Figure 7e). Similarly, after photodegradation treatment, the band is eliminated. The absorption band of Dye 1 (see Figure 7f) shows an absorption band around 382 nm–460 nm before photodegradation treatment. After photodegradation treatment, this band is reduced. The change of the fluorescence and the absorption indicates the degradation of the dyes, i.e., FSS and R6G. It should be noted that Dye 1 is the mixture of FSS and R6G. It is interesting to find out that it can be degraded by the nanoparticles.

Figure S2g,i–l reveal the color change of the dye solution before and after photodegradation treatment. The color of FSS is shifted from yellow to dark blue. The color of R6G is shifted from dark blue to black. The color of Dye 1 is shifted from dark green to yellow. The Ho-oxide compound showed photodegradation activities, which may be likely related to the effects of electron-hole recombination. The proposed mechanism of the photodegradation of the dyes is proposed as (Equations (1)–(6)): under white light-irradiation, the excited electrons and holes travel to the sample surface (Equation (1)) and their chemical reactions with surface species are generated. With the existence of the Ho-oxide compound, positive surface charges can be generated, leading to the formation of *OH^−^* ions attached to the surface of the dye material for charge balance. The attached *OH^−^* ions can accept holes to generate hydroxyl radicals, which are *OH* in Equation (3). The hole sites tend to combine with the attached dye molecules (Equation (2)). At the same time, the *^●^OH* radicals combine with the oxygenated structures (Equation (4)), leading to the formation of dye fragments. Finally, the dye fragments react with the active sites, including holes or *^●^OH* radicals to form *HCO_3_^−^*, *CO_3_^−^*, and *H^+^* (as shown in Equations (5) and (6)).

(1) $$Ho-oxide\;compound+photon\to hole^{+}+electron^{-}$$

(2) $$Dye_{attached}{}^{-}+hole^{+}\to HCO_{3}^{-}+CO_{3}^{2-}+3H^{+}$$

(3) OHattached−+hole+→OH●

(4) phenyl−O−R+OH●+H+→phenyl−OH+HO−R

(5) phenyl−OH+OH●→HCO3−+CO32−+3H+

(6) HO−R+OH●→HCO3−+CO32−+3H+

## 4. Conclusions

Ho-oxide compound nanoparticles were synthesized through facile chemistry. The nanoparticles were characterized by XRD, XPS, and TEM. They showed NIR fluorescence in the optical region of 800–1100 nm when an excitation light source with a center wavelength of 785 nm was used. Their potential in NIR animal-tissue imaging application was evaluated using the tissue of pig bacon meat. They present appealing tissue imaging applications with the maximum imaging depth below the tissue surface of 2.2 cm. Furthermore, the Ho-oxide compound is found to be useful for the degradation of FSS and R6G under white light irradiation. Our methodology for making these nanoparticles relies heavily on a solution synthesis approach. It may be cheaply and easily used in industry, where the normal hydrothermal synthesis used for autoclaves can lead to a high cost of fabrication. They show dual-functionalities in NIR animal-tissue imaging as well as dye-photodegradation.

## Data Availability

The data presented in this study are available in this article.

## Supplementary Materials

The following supporting information can be downloaded at: https://www.mdpi.com/article/10.3390/molecules27113522/s1, Figure S1: XRD profile. Its crystal information is derived to be Molecule formula: Ho_2_O_3_; Cell length: a = 18.8517 Å, b = 14.2007 Å, c = 12.9285 Å; lattice angle: α = 90˚, β = 109.74˚, γ = 90˚; symmetry: monoclinic; space group: P 21/n).; Figure S2: Bottles of dye solution before and after photodegradation treatment. g: FSS before photodegradation treatment. h: FSS after photodegradation treatment. i: R6G before photodegradation treatment. j: R6G after photodegradation treatment. k: Dye1 before photodegradation treatment. l: Dye1 after photodegradation treatment.; Table S1: Molecular structure, toxicity, and molecular mass of dyes.

## Sample Availability

Samples of the compounds are available from the authors upon request.
