# Supplementary material for: Synthesis of Holmium-Oxide Nanoparticles for Near-Infrared Imaging and Dye-Photodegradation
Source: Molecules. 2022 May 30;27(11):3522. doi: 10.3390/molecules27113522 (PMC9181859; doi:10.3390/molecules27113522)
Supplement: Supplementary file 1 [file molecules-27-03522-s001.zip › molecules-1702208-supplementary.pdf]

molecules

MDPI

Supplementary Materials

Synthesis of Holmium-Oxide Nanoparticles for Near-Infrared Imaging and Dye-Photodegradation

Jia Zhu [1,†], Xue-Jun Shao [2,†], Zongan Li [3], Chia-Hui Lin [4], Cheng-Wan-Qian Wang [5], Keran Jiao [6], Jian Xu [7,*], Hong-Xia Pan [5,*] and Ye Wu [3,*]

1 Department of Mechatronic Engineering, Suzhou City University, Suzhou, ,215000, China; jia.zhu@szcu.edu.cn
2 Department of Clinical Laboratory Medicine, Children's Hospital of Soochow University, Suzhou, 215025, China; xjshao@suda.edu.cn
3 Jiangsu Key Laboratory of 3D Printing Equipment and Manufacturing, School of Electrical and Automation Engineering, Nanjing Normal University, Nanjing, 210046, China; zongan_li@njnu.edu.cn
4 Suzhou Gallant Biotech Biotechnology Co. Ltd., Suzhou, 215000, China; calacolin@gpmbio.cn
5 Department of Clinical Laboratory Medicine, Suzhou BenQ Medical Center, Affiliated BenQ Hospital of Nanjing Medical University, Suzhou, 215000, China; wanqian_1019@163.com
6 Department of Chemistry, Xi'an Jiaotong-Liverpool University, Suzhou, 215000, China; keran.jiao16@student.xjtlu.edu.cn
7 Division of Electrical and Computer Engineering, Louisiana State University, Baton Rouge, LA 70803, USA
* Correspondence: jianxu1@lsu.edu (J.X.); panhongxiajyk@163.com (H.-X.P.); chemwuye@njnu.edu.cn (Y.W.)
† These authors contributed equally to this work.

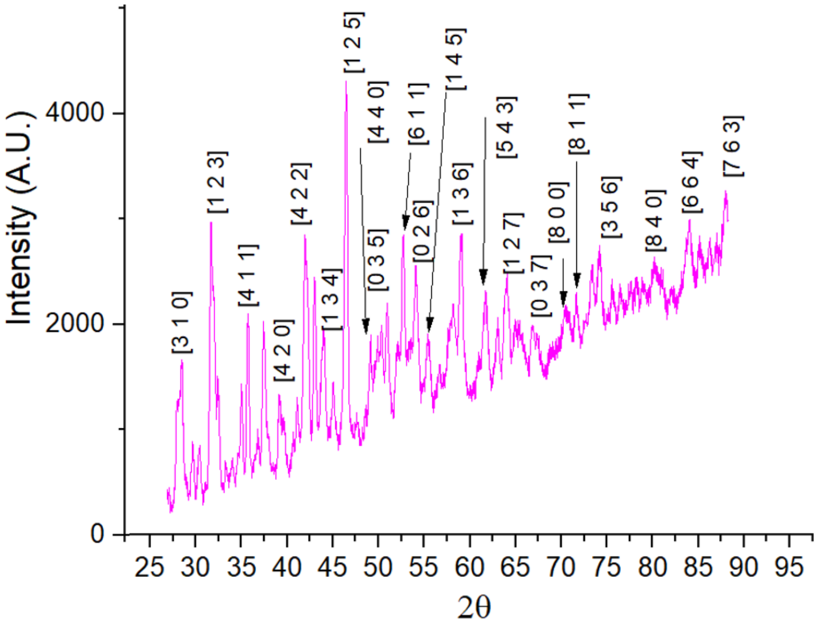

Figure S1. XRD profile. Its crystal information is derived to be Molecule formula: Ho_2O_3; Cell length: a = 18.8517 Å, b = 14.2007 Å, c = 12.9285 Å; lattice angle: α = 90°, β = 109.74°, γ = 90°; symmetry: monoclinic; space group: P 21/n).

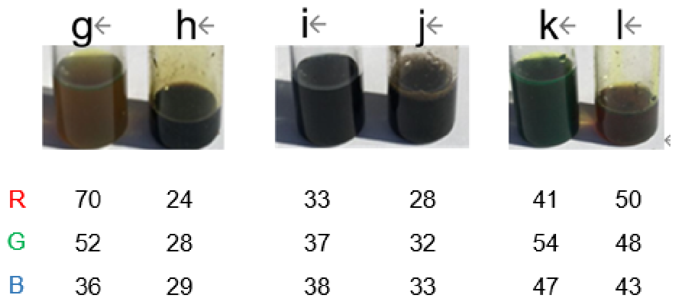

Figure S2. Bottles of dye solution before and after photodegradation treatment. g: FSS before photodegradation treatment. h: FSS after photodegradation treatment. i: R6G before photodegradation treatment. j: R6G after photodegradation treatment. k: Dye1 before photodegradation treatment. l: Dye1 after photodegradation treatment.

Table S1. Molecular structure, toxicity, and molecular mass of dyes.

	**Fluorescein Sodium Salt**	**Rhodamine 6G**
Molecular Formula	$C_{20}H_{10}Na_2O_5$	$C_{28}H_{31}N_2O_3Cl$
Molecular Structure	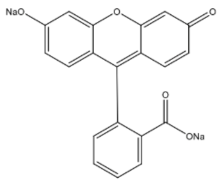	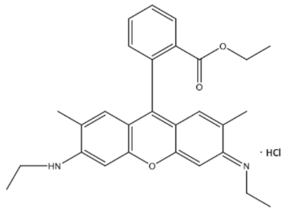
Molecular Mass	376.27	479.01
Toxicity	Acute toxicity: LD_{50} Oral-Rat-6720 mg/kg	Acute toxicity, Oral
